# Study on the regulatory role of MINK1 gene in the activation of NLRP3 inflammasome in common carp (*Cyprinus carpio* L.)

**DOI:** 10.3389/fimmu.2025.1663527

**Published:** 2025-10-22

**Authors:** Rongrong Liu, Keying Zhao, Yue Zhao, Guiwen Yang, Hua Li

**Affiliations:** Shandong Provincial Key Laboratory of Animal Resistance Biology, College of Life Sciences, Shandong Normal University, Jinan, China

**Keywords:** common carp, MINK1, expression patterns, anti-infectious immunity, NLRP3 inflammasome

## Abstract

The nucleotide oligomerization domain (NOD)-like receptor protein 3 (NLRP3) inflammasome is a cytosolic multiprotein complex that can be activated by a wide variety of stimuli. However, dysregulated activation of NLRP3 is implicated in the pathogenesis of chronic inflammatory diseases. Hence, the activity of NLRP3 is intricately governed by several regulatory mechanisms. Misshapen-like kinase 1 (MINK1), a serine/threonine kinase, plays an important role in the immune cell differentiation and inflammatory response regulation in mammals; however, its regulatory function in NLRP3 inflammasome activation in fish remains poorly understood. In the present study, a homolog gene of MINK1 (*Cc*MINK1) was cloned and functionally characterized in common carp (*Cyprinus carpio* L.). The expression profiling disclosed that *CcMINK1* was upregulated under spring viremia of carp virus (SVCV) and *Aeromonas hydrophila* stimulation. Overexpression of *Cc*MINK1 promoted *Cc*NLRP3-mediated inflammasome activation, including apoptosis-associated speck-like protein containing a CARD (ASC) oligomerization, speck formation, cysteine-requiring aspartate protease A/B (Caspase-A/B) enzyme activity and interleukin-1β (IL-1β) cleavage. Mechanistically, *Cc*MINK1 interacted with *Cc*NLRP3 via its S_TKC domain and facilitated *Cc*NLRP3 phosphorylation, thereby promoting its aggregation and activation. Collectively, these discoveries unveil a novel regulatory mechanism that governs the functional regulation of *Cc*NLRP3 and fine-tuning innate immune responses in teleost.

## 1 Introduction

In innate immune system, the pattern recognition receptors (PRRs) are used to detect invading pathogens and endogenous damager signal, thereby initiating signaling cascades that maintain homeostasis (1). Currently, the PRRs that have been identified are mainly classified into four categories: NOD-like receptors (NLRs), Toll-like receptors (TLRs), RIG-I-like receptors (RLRs), and C-type lectin receptors (CLRs) (2–5). Among them, NLRs represent a highly versatile family of receptors capable of recognizing diverse ligands and modulating critical immune processes, including inflammasome assembly, apoptosis, and other immune signaling pathways (6). A variety of inflammasomes were reported in mammals, with the nucleotide oligomerization domain (NOD)-like receptor protein 3 (NLRP3) inflammasome being the most extensively characterized (7). The activation of NLRP3 inflammasome is a tightly regulated, two-step process involving priming and activation (8). Activated NLRP3 recruits ASC to form the inflammasome complex, and then recruits pro-Caspase-1, which cleaves pro-interleukin-1β (IL-1β) and pro-interleukin-18 (IL-18) to mature IL-1β and IL-18 (9, 10). Additionally, activated Caspase-1 also mediated the cleavage of gasdermin D (GSDMD), generating N-terminal fragments that translocate to the plasma membrane to induce pyroptosis, a lytic and inflammatory form of programmed cell death (11–13).

The dysregulated activation of the NLRP3 inflammasome has been implicated in the pathogenesis of numerous metabolic- and aging-associated inflammatory disorders, such as gout, atherosclerosis, Alzheimer’s disease and cytokine storm (14–17). Consequently, precise modulation of NLRP3 activation is critically important for maintaining immune homeostasis. Several posttranslational modifications (PTMs) have been reported to regulate NLRP3 inflammasome activation by influencing protein stability, subcellular localization, ATPase activity and interactions with other inflammasome components (18, 19). For instance, palmitoylation of NLRP3 by zinc finger DHHC-type palmitoyl transferase 7 (ZDHHC7) facilitates its localization to the trans-Golgi network (TGN), a prerequisite for subsequent ASC recruitment and oligomerization (20). Similarly, membrane associated ring-CH-type finger 5 (MARCH5), a mitochondria-associated E3 ligase, mediates K27-linked polyubiquitination of NLRP3, enhancing its role in antimicrobial immunity (21). Additionally, the E3 SUMO ligase TRIM28 binds NLRP3 and promotes its SUMOylation, thereby inhibiting proteasomal degradation and facilitating NLRP3 inflammasome assembly (22). AKT phosphorylates NLRP3 on Ser5, and further stabilizes NLRP3 and restrict its oligomerization (23). Furthermore, acetylation of NLRP3 by the lysine acetyltransferase 5 (KAT5) is important for its oligomerization and functional inflammasome formation (24). Although these regulatory mechanisms of NLRP3 are well-established in mammals, the corresponding regulatory factors and PTMs in lower vertebrates such as teleost fish are largely unexplored.

Misshapen/Nck-interacting kinase–related kinase 1 (MINK1) is an evolutionarily conserved Ste20-like serine/threonine kinase that belongs to the germinal center kinase (GCK) IV family (25). MINK1 plays crucial roles in a variety of biological processes, such as cell-matrix adhesion, platelet function, and cytokinesis (26, 27). Furthermore, MINK1 also plays a critical role in the activation of c-Jun N-terminal kinase (JNK) and Ras-mediated p38 MAP kinase pathways, thereby regulating cellular senescence, cytoskeletal organization and cell motility (25, 28). Zhu et al. recently have identified that MINK1 significantly contributes to NLRP3 inflammasome activation and assembly by promoting phosphorylation of NLRP3 at Ser725 (29). While mammalian MINK1 has been extensively characterized for its roles in cancer progression, cell fate determination, and innate immunity, the functional significance of MINK1 in teleost remains largely unexplored.

As lower vertebrates, bony fish mainly rely on the innate immune system to defend against the invasion of pathogenic microorganisms (30). However, the molecular mechanisms underlying inflammasome assembly in fish remain poorly characterized. In the present study, we identified that common carp MINKI (*Cc*MINK1) serves as a positive regulator of inflammasome activation. The mechanistic investigations revealed that *Cc*MINK1 physically interacted with and phosphorylated *Cc*NLRP3, thereby leading to its activation. These findings provide novel insights into the molecular mechanisms of inflammasome regulation in teleost and suggest that MINK1 may represent a promising therapeutic target for managing viral infections in aquaculture species.

## 2 Materials and methods

### 2.1 Cells and transfection

293T were obtained from the Cell Bank of the Chinese Academy of Sciences, while Epithelioma papulosum cyprini (EPC) cells were provided by the Freshwater Fisheries Research Institute of Shandong Province. 293T cells were cultured in DMEM medium, which was added with 10% fetal bovine serum (FBS), 10 units/ml penicillin and 10 mg/mL streptomycin at 37 °C in a 5% CO_2_ humidified atmosphere. EPC cells were cultured in M199 medium containing 10% FBS, 1% streptomycin and penicillin at 25 °C. Transient transfections in 293T and EPC cells were carried using Lipofectamine 2000 (Invitrogen, USA) and jetPRIME reagent (Polyplus, France) following the manufacturers’ protocols, respectively.

### 2.2 Fish raising and pathogen challenge

The common carp (about 200 g per fish) were purchased from commercial pond farms in Jinan, China. The fish were cultured in recirculating water (25 ± 2°C) for a minimum of seven days before the experiment and fed twice a day. For tissue expression analysis, three healthy carp with similar size and age were selected and dissected. Ten different tissues, including the liver, foregut, hindgut, brain, skin, head kidney, gills, muscle, heart, and spleen were sequentially acquired. Additionally, in pathogen stimulation group, 500 μL of SVCV (10^4.8^ TCID50/100 μL) or *A. hydrophila* (2 × 10^8^ CFU/ml) were intraperitoneally injected into carp, and the control group was injected with the same volume of M199 medium or PBS. After infection indicated times, the immune-related tissues (liver, spleen, foregut, and hindgut) were collected and immediately storaged at -80°C for subsequent analysis.

### 2.3 The bioinformatics analysis of *Cc*MINK1

The MINK1 gene sequence from common carp was retrieved from the Ensembl genomes database and subsequently cloned using standard molecular biology. The nucleic acid sequence of *Cc*MINK1 were translated into amino acid sequence using BioEdit software and then protein structure of *Cc*MINK1 was predicted using SMART analysis (http://smart.embl-heidelberg.de/). The three-dimensional (3D) structure of MINK1 was further predicted via SWISS-MODEL analysis (https://swissmodel.expasy.org/). For comparative analysis, the MINK1 protein sequence across multiple species was obtained from the NCBI database. The phylogenetic tree was constructed using MEGA7.0 software with the neighbor-joining method. The GenBank accession numbers of all analyzed sequences are provided in **Table 1**.

**Table 1:** The GenBank accession numbers of the MINK1 in different species.

Species	Genbank accession numbers
*Homo sapiens*	NP_056531.1
*Pan troglodytes*	XP_054527063.1
*Sus scrofa*	XP_020923447.1
*Mus musculus*	NP_795712.2
*Oryctolagus cuniculus*	XP_051681266.1
*Carassius carassius*	XP_059406383.1
*Cyprinus carpio*	XP_042580329.1
*Cyprinodon variegatus*	XP_015253531.1
*Paralichthys olivaceus*	XP_019941351.1
*Chelonia mydas*	XP_037743240.1
*Ctenopharyngodon idella*	XP_051749122.1
*Puntigrus tetrazona*	XP_043096321.1
*Megalobrama amblycephala*	XP_048044071.1
*imephales promelas*	XP_039515271.1
*Astyanax mexicanus*	XP_022534311.1
*Danio aesculapii*	XP_056314298.1

### 2.4 Plasmid construction

The eukaryotic expression vector encoding *Cc*MINK1-GFP, *Cc*MINK1-Myc, *Cc*MINK1-HA and *Cc*MINK1-Flag were generated using standard molecular biology as described previously (31). In brief, the ORF of *Cc*MINK1 was amplificated and directionally cloned into indicated vector using T4 DNA ligase. Then, the recombinant plasmid was confirmed by TSINGKE Biological Technology and the correct expression vectors were extracted through the Endo-Free Plasmid Mini Kit II (Omega Bio-Tek). The primers used in this study are provided in **Table 2**.

**Table 2:** The primers used in this study.

Names	Sequences (5’-3’)
*Cc*MINK1-HA-F	CCGGAATTCGGATGTCAGAAAACGCACCTAC
*Cc*MINK1-HA-R	CGGGGTACCTCACCAGTTCATAATGCAGTT
*Cc*MINK1-Myc-F	CCGGAATTCCGCCACCATGTCAGAAAACGCACCTAC
*Cc*MINK1-Myc-R	CGGGGTACCCCAGTTCATAATGCAGTTTCT
*Cc*MINK1-Flag-F	GCCACCATGGACTACCCTGCAGGAATGTCAGAAAACGCACCTACG
*Cc*MINK1-Flag-R	CTTGTCATCATCGTCGCTAGCTCACCAGTTCATAATGCAGTT
*Cc*MINK1-GFP-F	CTCGAGCTCAAGCTTCGAATTCTATGTCAGAAAACGCACCTACG
*Cc*MINK1-GFP-R	GGATCCCGGGCCCGCGGTACCTCACCAGTTCATAATGCAGTT
S11-QF	CCGTGGGTGACATCGTTACA
S11-QR	TCAGGACATTGAACCTCACTGTCT
*Cc*MINK1-QF	GAGGTGGTGGGTAA
*Cc*MINK1-QR	GGCTCCGTAGTAGG

### 2.5 RNA extraction and qPCR

Total RNA from tissues samples and cells were isolated via using either RNA simple Total RNA kit (Nobelab) or Trizol reagent following the manufacturers’ protocols. Then, the a Fast Quant Kit (with gDNase) (Nobelab) was used for the reverse transcription of RNA, in line with the manufacturer’s protocol. 2 × SYBR Premix UrTaq II (Nobelab) were applied for the qPCR assay using the LightCycler 96 instrument (Roche). The thermal cycling protocol commenced with an initial step at 95 °C for 120 s, succeeded by 40 cycles of 95 °C for 10 s and annealing/extension at 60 °C for a duration of 30 s. The gene expression levels were normalized to S11 and EF-1α expression, respectively. The details of primers used in the present study are described in **Table 2**.

### 2.6 Co-immunoprecipitation and immunoblotting

293T cells were transfected with indicated expressing vectors on the figure. After 48 h, the cells lysates were generated on ice using 1%NP-40 lysis buffer supplemented with proteinase inhibitors. For immunoprecipitation assays, cells lysates were incubated with the appropriate affinity gel overnight at 4 °C with gentle rotation. Then, beads were subsequently washed four times with ice-cold 1%NP-40 lysis buffer, the bound proteins were eluted in 2×SDS loading buffer. The whole-cell extracts and ip samples were used for immunoblotting assays.

For immunoblotting analysis, protein was resolved by indicated SDS-PAGE gels and transferred onto PVDF membranes through standard protocols. Membranes were blocked with 5% nonfat milk prior to incubation with primary antibodies: GFP (HUABIO, ET1607-31, 1:20000), HA (Abcam, ab9110, 1:1000), Myc (CST, 2278T, 1:1000) and Flag (Solarbio, K200001M, 1:10000) overnight under 4 °C, respectively. Subsequently, the membrane was washed four using 1×TBST (TBS encompassing 0.1% Tween-20) and incubated with the appropriate horseradish peroxidase (HRP)-conjugated secondary antibodies (Jackson immunoresearch, USA). Protein band was visualized using Enhanced Chemiluminescence Detection Reagents (Meilunbio, China).

### 2.7 Immunofluorescence assay

293T or EPC cells were cultured in a 24-well plate with glass slides and transfected with the indicated expression plasmid for 24 h. The cells were washed with PBS and fixed with 4% paraformaldehyde (PFA) for 30 min. Then, the cells were washed with PBS and DAPI (Beyotime, C1002, 1:1000) was used to stain the nuclear. The stained cells were viewed using a Leica SP8 confocal laser scanning microscope.

### 2.8 Caspase-A/B enzyme activity detection

Several previous studies have shown that there are two homologs of proinflammatory Caspase, namely Caspase-A and Caspase-B in zebrafish (32–34) and common carp (35). In particular, two transcripts of Caspase-A genes were cloned from common carp, which were named *Cc*Caspase-A1 and *Cc*Caspase-A2 (35). 293T cells were cultured in a 24-well plate and transfected with the indicated expression plasmid for 24 h. The *Cc*Caspase-A1/A2/B enzyme activity was detected using the enzyme activity detection kit (Enzo Life Sciences, USA) and the fluorescence value was detected by multifunctional enzyme-linked immunosorbent assay instrument (FilterMax F3, Austria). Caspase-A1/A2/B enzyme activity = (fluorescence value of experimental group - fluorescence value of control group)/fluorescence value of control group×100%.

### 2.9 Cytotoxicity assay

LDH cytotoxicity was quantified using the CytoTox 96^®^ Non-Radioactive Cytotoxicity Assay Kit (Promega). Briefly, 293T cells were seeded in 48-well plates and transfected with corresponding plasmids for 48 h. Following transfection, cells were treated with 10×Lysis Solution for 45 min at 37 °C as positive control. For assay measurements, 33 μL of cell supernatant was transferred to 96-well plate and mixed with an equal volume of reconstituted CytoTox 96^®^ Reagent. After 30 minutes of protected incubation at room temperature, the reaction was terminated by adding 33 μL of Stop Solution. Absorbance at 492 nm was measured through a FilterMax F3 Multi-Mode Microplate Reader. Cytotoxicity (%) was calculated as experimental LDH release/maximum LDH release × 100%, with background subtraction using untreated cells.

### 2.10 ASC oligomerization assay

293T cells were transfected with indicated expression plasmids. After 48 h, the cells was lysed and centrifuged at 4 °C, 6000 × g for 5 min. The supernatant was kept for immunoblotting as loading control and the pellet was washed with PBS twice and cross-linked with 2 mM DSS for 30 min at room temperature. After centrifuging at 4 °C, 5000 × g for 10 min, the pellet was resuspended with 50 μL SDS-PAGE loading buffer and determined by immunoblotting analysis with the indicated antibodies.

### 2.11 Phos-tag SDS-PAGE

Phos-tag gels were purchased from FUJIFILM Wako Pure Chemical Corporation and carried out by the same way as immunoblotting assays, except that (1): The Phos-tag gels were soaked in EDTA for 10 min before transfer onto a PVDF membrane to remove Zn^2+^ (2): The Phos-tag gels were soaked transmembrane buffer solution containing 10 mmol/L EDTA and washed twice.

### 2.12 Statistical analysis

All data are presented as the mean ± standard deviation (SD) from three independent experiments. Data were analyzed using GraphPad Prism 8. Comparisons between two groups were analyzed using the Student’s *t* test. P-value less than 0.05 was considered statistically significant.

## 3 Results

### 3.1 The bioinformatics analysis of *Cc*MINK1

The ORF of MINK1 were cloned and identified from the spleen of common carp, which consisted of 3687 bp and encoded 1229 amino acids. Domain architecture prediction using the SMART online tool demonstrated that *Cc*MINK1 contained a N-terminal S_TKc domain and a C-terrmina CNH domain (**Supplementary Figure 1A**). Subsequently, three-dimensional (3D) architectures modeling of MINK1 proteins across vertebrate species was contrasted using SWISS-MODEL, which showed that the 3D structure of *Cc*MINK1 was similar to that of other species (**Supplementary Figure 1B**). Additionally, the phylogenetic tree was built to investigate the evolutionary relationship of MINK1, which suggested that *Cc*MINK1 was closely related to that of *Carassius auratus* (**Supplementary Figure 1C**).

### 3.2 The subcellular localization and tissue distribution of *Cc*MINK1

**Figure 1:**
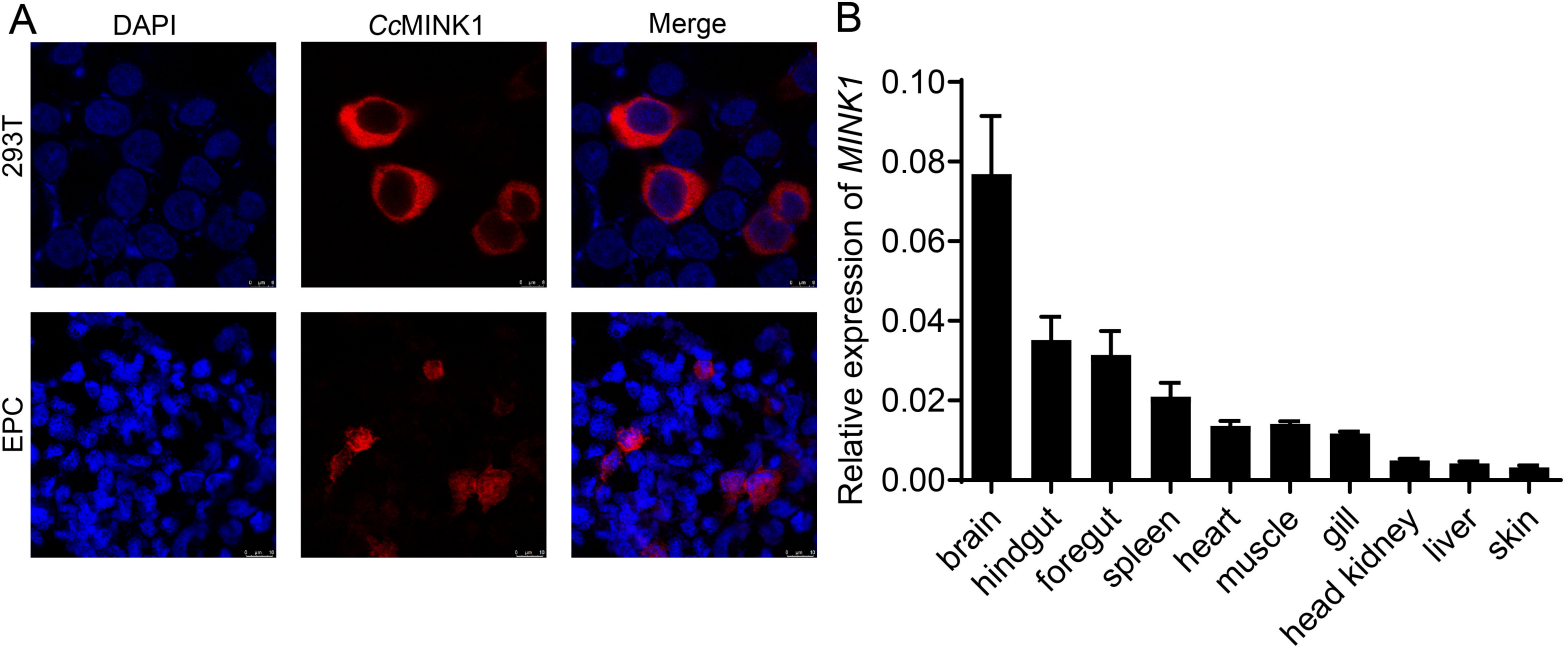
The subcellular localization and tissue expression of *Cc*MINK1. **(A)** 293T cells or EPC cells were seed onto 24-well coverslips and transfected with *Cc*MINK1 plasmid with mCherry-tag. After 24 h, the cells were fixed with 4% paraformaldehyde (PFA) for 30 min. Then, the cells were washed with PBS and DAPI was used to stain the nuclear. The cells observed with confocal microscopy. Red indicates the *Cc*MINK1. Blue represents the nucleus. **(B)** The mRNA expression levels of *CcMINK1* in ten tissues of healthy common carp were determined by qPCR. The common carp 40S ribosomal protein (S11) was used as an internal reference, mean ± SD (n = 3).

To investigate the biological properties of *Cc*MINK1, the subcellular localization of *Cc*MINK1 was evaluated. The recombinant plasmid *Cc*MINK1-mCherry was transfected into 293T and EPC cells. Confocal microscopy analysis revealed that *Cc*MINK1 was exclusively expressed in the cytoplasm of both cell lines (**Figure 1A**). Subsequently, we further delineate the mRNA expression profiles of *CcMINK1* in the brain, hindgut, foregut, spleen, heart, muscle, gills, head kidney, liver and skin. qPCR results showed that *CcMINK1* was universally expressed across all investigated tissues, and the expression was substantially higher in the brain and hindgut, followed by the foregut and spleen, while skin showed the lowest expression among all tested tissues (**Figure 1B**).

### 3.3 The expression profiles of *CcMINK1* under various immune stimulation

**Figure 2:**
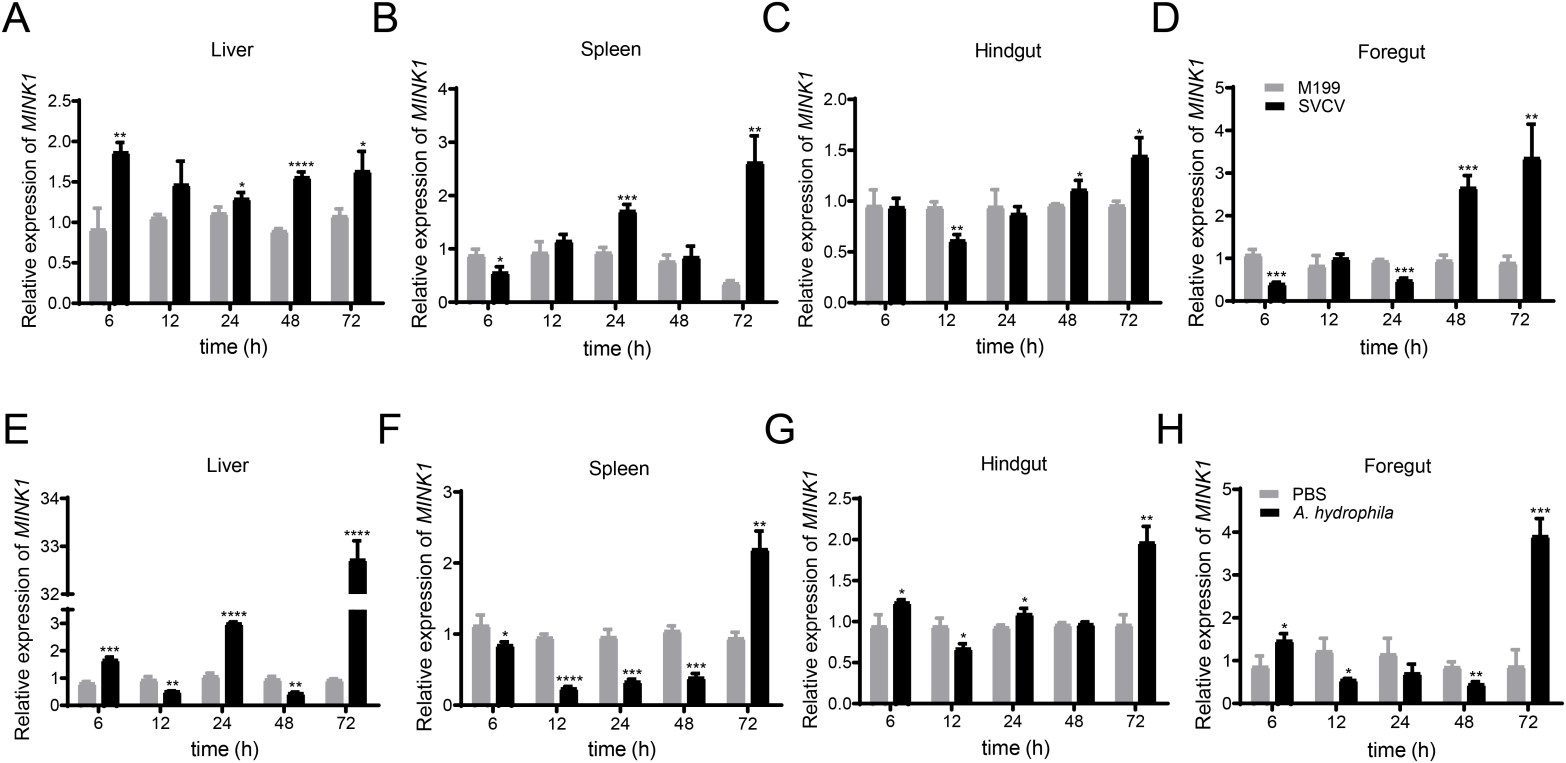
The mRNA expression patterns of *CcMINK1* in response to spring viremia of carp virus (SVCV) and *Aeromonas hydrophila* (*A. hydrophila*) stimulation in different tissues of common carp. Upon stimulation with SVCV and *A. hydrophila*, the transcriptional change patterns of *CcMINK1* in liver **(A, E)**, spleen **(B, F)**, hindgut **(C, G)** and foregut **(D, H)** at different time points were detected by qPCR. M199 or PBS treatment was used as a control. The data are normalized to S11 gene expression and are displayed as the mean ± SD (n=3), *P < 0.05, **P < 0.01, ***P < 0.001, ****P < 0.0001.

To examine the *CcMINK1* expression profiles following pathogen infection, several immune-related tissues were extracted from the common carp at specified time points after SVCV or *A. hydrophila* infection. Under the stimulation of SVCV, the expression of *CcMINK1* in various tissues of common carp significantly increased (**Figure 2**). In the liver, the *CcMINK1* mRNA levels were up-regulated and reached a peak value at 6 h (about 2.0-fold) (**Figure 2A**). In the spleen, hindgut and foregut, *CcMINK1* transcript levels initially displayed a decrease at 6 h, then start to increase at 24 h and 48 h, and achieved the highest at 72 h, respectively (**Figures 2B–D**). Similarly, under the infection of *A. hydrophila*, the *CcMINK1* expression level was observed to be increased at indicated points in all examined tissues. In the liver, foregut and hindgut, the *CcMINK1* mRNA was induced at 6 h, and arrived the highest level at 72 h (**Figures 2E, G, H**). In the spleen, *CcMINK1* expression levels initially displayed a decrease, and achieved the highest at 72 h (**Figure 2F**). In summary, these above results illustrate that *Cc*MINK1 might response to both antiviral and antibacterial immune infection of common carp.

### 3.4 *Cc*MINK1 interacts with *Cc*NLRP3

**Figure 3:**
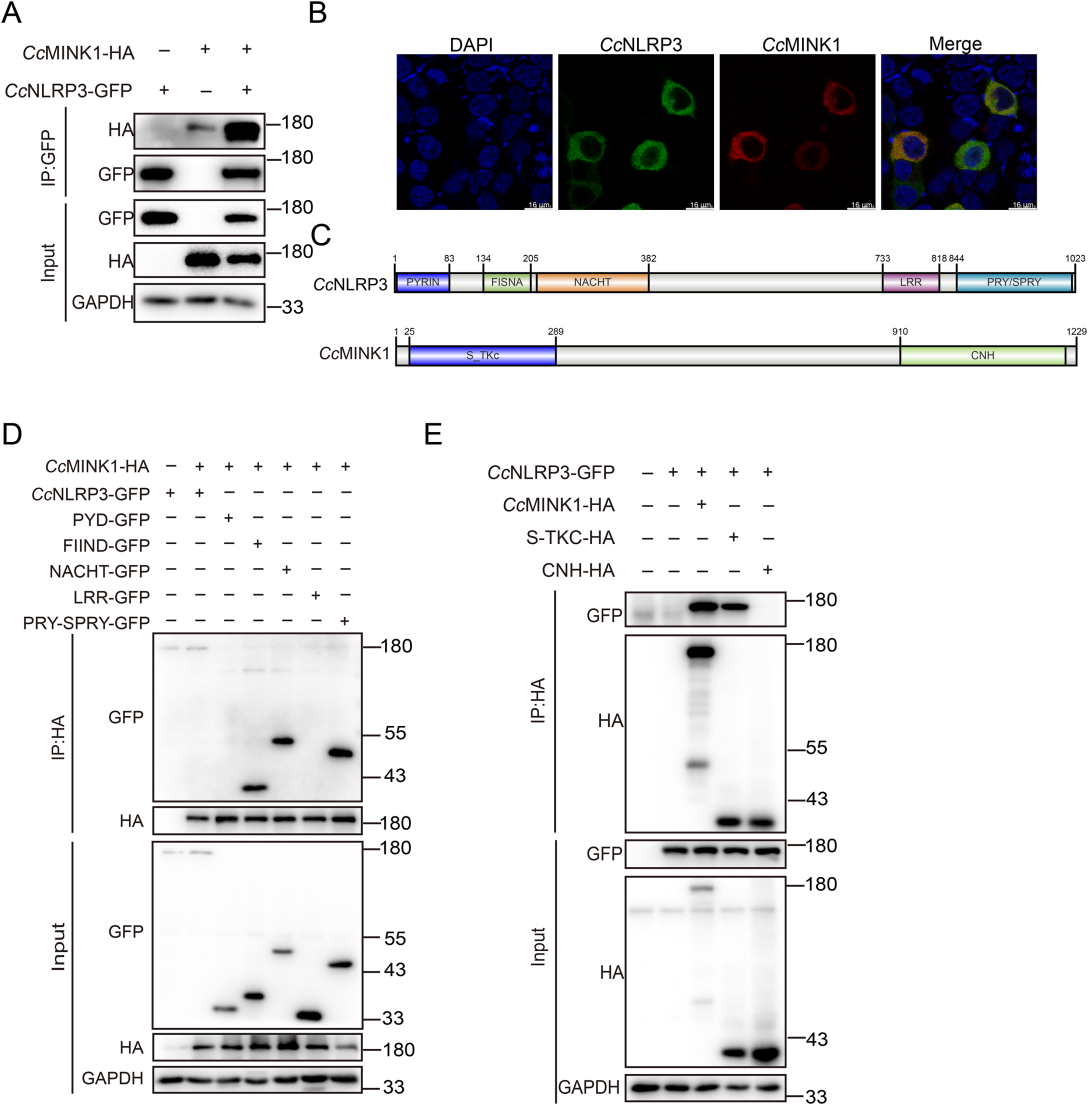
*Cc*MINK1 associates with *Cc*NLRP3. **(A)** 293T cells were transfected with *Cc*MINK1-HA and *Cc*NLRP3-GFP expression plasmids. At 48 h, immunoprecipitation was performed with anti-GFP beads, and the input and IP were analyzed by immunoblot assays. **(B)** 293T cells were transfected with *Cc*MINK1-mCherry and *Cc*NLRP3-GFP expression plasmids. After 24 h, the cells were fixed with 4% paraformaldehyde (PFA) for 30 min. Then, the cells were washed with PBS and DAPI was used to stain the nuclear. The cells observed with confocal microscopy. **(C)** Schematic diagram of full-length *Cc*NLRP3 and *Cc*MINK1 along with its domain expression vectors. **(D)** 293T cells were transfected with *Cc*MINK1-HA and *Cc*NLRP3-GFP or its domain expression plasmids, respectively. At 48 h, immunoprecipitation was performed with anti-HA beads, and the input and IP were analyzed by immunoblot assays. **(E)** 293T cells were transfected with *Cc*NLRP3-GFP and *Cc*MINK1-HA or its domain expression plasmids, respectively. At 48 h, immunoprecipitation was performed with anti-HA beads, and the input and IP were analyzed by immunoblot assays.

Previous studies have shown that mammals MINK1 interacts with NLRP3 to regulate the assembly of NLRP3 inflammasome (29), while its role in teleost is unclear. To explain the role of *Cc*MINK1 in the regulation of *Cc*NLRP3, we firstly examined the interaction of *Cc*MINK1 and *Cc*NLRP3. Co-ip experiments revealed that *Cc*MINK1 could interact with *Cc*NLRP3 in 293T cells (**Figure 3A**). Similarly, we found that *Cc*MINK1 colocalized with *Cc*NLRP3 in the cytoplasm by confocal microscopy (**Figure 3B**). To further identify which domain of *Cc*MINK1 is responsible for its interaction with *Cc*NLRP3, the recombinant plasmids expressing different domains of *Cc*NLRP3 and *Cc*MINK1 were generated separately (**Figure 3C**). The results of Co-ip revealed that the FIIND, NACHT and PRY-SPRY domain of *Cc*NLRP3 interacted with *Cc*MINK1, but its PYD and LRR domain showed no interaction with *Cc*MINK1 (**Figure 3D**). Additionally, the S_TKC domain of *Cc*MINK1 interacted with *Cc*NLRP3, whereas its CNH domain showed no interaction with *Cc*NLRP3 (**Figure 3E**). Collectively, these results suggested that the S_TKC domain of *Cc*MINK1 directly targeted with the FIIND, NACHT and PRY-SPRY domain of *Cc*NLRP3.

### 3.5 *Cc*MINK1 promotes *Cc*NLRP3-mediated *Cc*ASC oligomerization and specks formation

**Figure 4:**
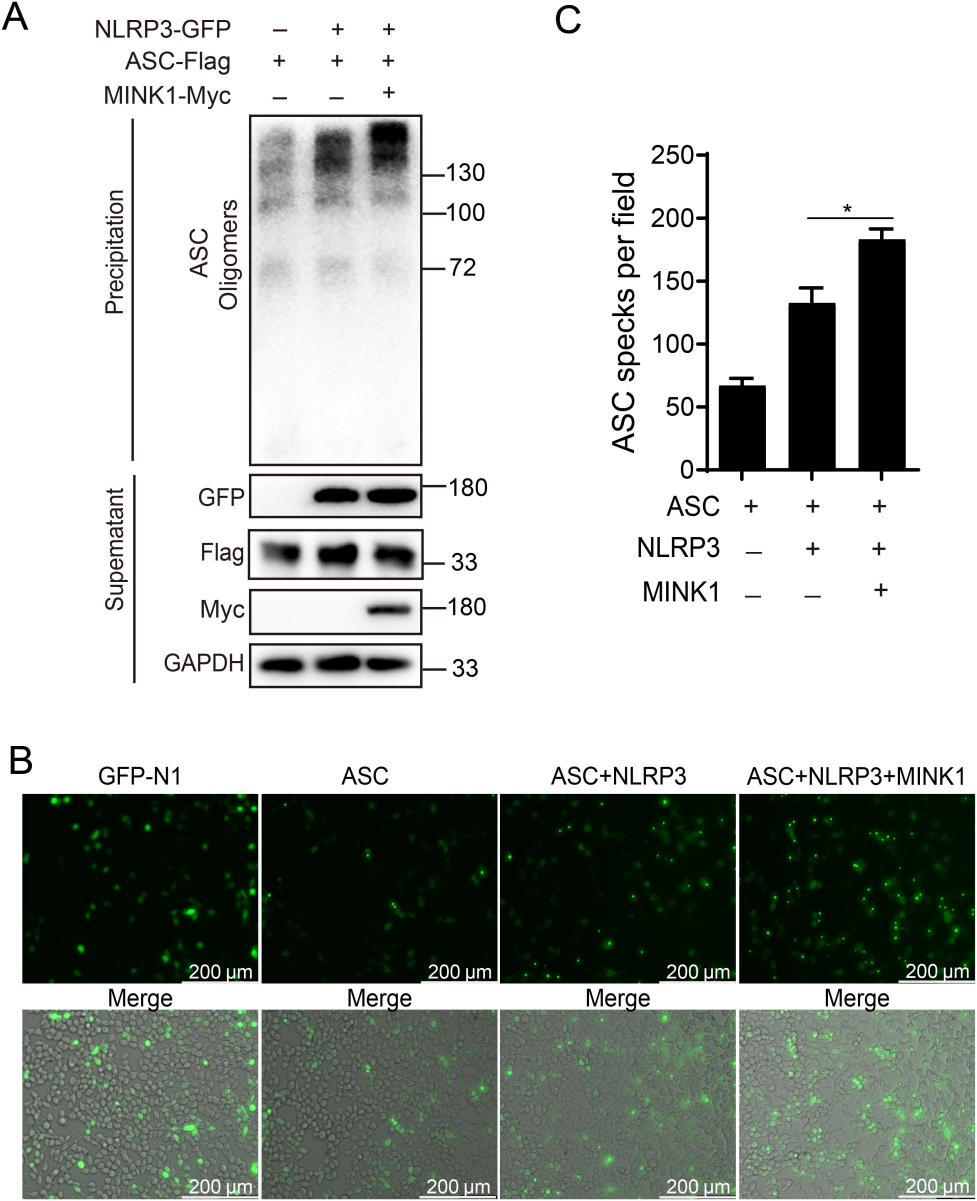
*Cc*MINK1 boosts the *Cc*ASC oligomerization and specks formation mediated by *Cc*NLRP3. **(A)** 293T cells were subjected to transfection with *Cc*MINK1-Myc, *Cc*ASC-Flag and *Cc*NLRP3-GFP. After 48 h, the cells was lysed and centrifuged. The supernatant was kept for immunoblotting as loading control and the pellet was washed with PBS twice and cross-linked with 2 mM DSS for 30 min at room temperature. After centrifuging, the pellet was resuspended with 50 μL SDS-PAGE protein loading buffer and determined by immunoblotting analysis with the indicated antibodies. **(B)** 293T cells were transfected with the indicated plasmids. 24 hours post transfection, the cells were observed and the images were visualized by confocal microscopy. **(C)** The relative mean value of *Cc*ASC specks in per field were calculated by ImageJ. Data were emerged as mean ± SD (n=3) with at least three replicates, *P < 0.05.

Activated NLRP3 recruits downstream adaptor ASC through the CARD domain, and then ASC assembles into a large protein complex, or “speck”, that is considered to be an upstream readout for inflammasome activation (9). Thus, to determine the functions of *Cc*MINK1 in activation of *Cc*NLRP3 inflammasome, *Cc*MINK1 was co-transfected with *Cc*NLRP3 and *Cc*ASC into 293T cells, and DSS crosslink analysis was used to explore the effect of *Cc*MINK1 on *Cc*ASC oligomerization. As shown in **Figure 4A**, *Cc*NLRP3 overexpression markedly boosted the oligomerization of *Cc*ASC, and more oligomerization was observed in *Cc*MINK1 overexpressed 293T cells. In addition, immunofluorescence staining showed that *Cc*MINK1 significantly enhanced the speck formation of *Cc*ASC mediated by *Cc*NLRP3 both in 293T and EPC cells (**Figures 4B, C**, **Supplementary Figures 2A, B**). Taken together, these results indicate that *Cc*MINK1 promotes *Cc*NLRP3-mediated the *Cc*ASC oligomerization and specks formation.

### 3.6 *Cc*MINK1 promotes *Cc*NLRP3-dependent *Cc*Caspase-1 activity and *Cc*IL-1β production

**Figure 5:**
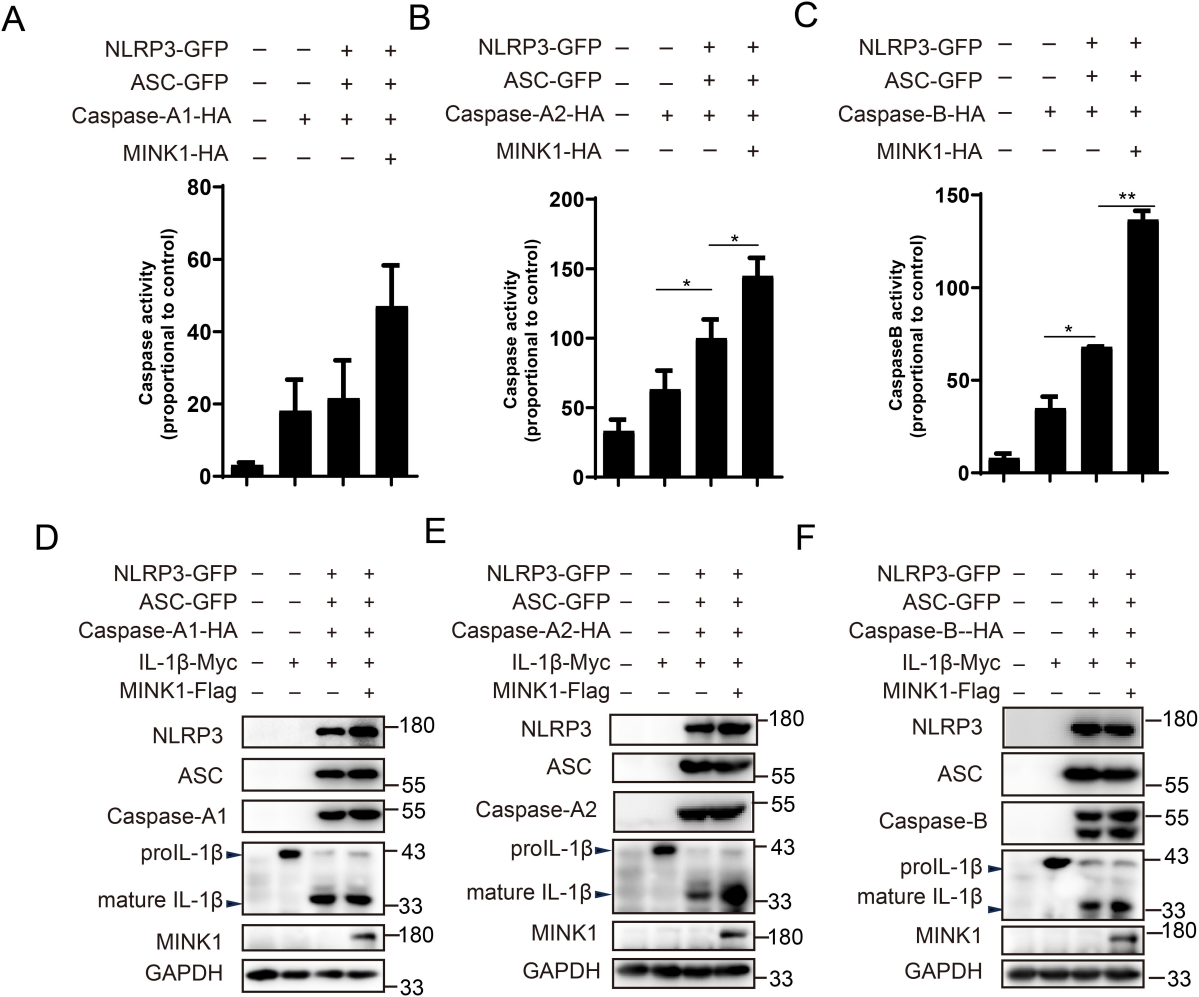
*Cc*MINK1 promotes *Cc*NLRP3-dependent *Cc*Caspase-1 activity and *Cc*IL-1β production. **(A–C)** 293T cells were transfected with *Cc*NLRP3-GFP, *Cc*ASC-GFP, *Cc*Caspase-A1/A2/B-HA and *Cc*MINK1-HA. After 48 h, the activity of *Cc*Caspase-A1, *Cc*Caspase-A2 and *Cc*Caspase-B were examined. **(D–F)** 293T cells were transfected with *Cc*NLRP3-GFP, *Cc*ASC-GFP, *Cc*Caspase-A1/A2/B-HA, *Cc*IL-1β-Myc and *Cc*MINK1-Flag. After 48 h, the protein was extracted and immunoblot assays was used to examined the expression of indicated protein. Data were emerged as mean ± SD (n=3) with at least three replicates, *P < 0.05, and **P < 0.01.

After being activated, the NLRP3 recruit pro-Caspase-1 via ASC and cleave it to active Caspase-1, which further cleaves pro-IL-1β and GSDMD to mature forms (36). Thus, we investigated whether *Cc*MINK1 modulates the activity of *Cc*Caspases. As shown in **Figures 5A–C**, *Cc*NLRP3 significantly boosted the *Cc*Caspase-A2 and *Cc*Caspase-B activity, and *Cc*MINK1 overexpressed further enhanced *Cc*NLRP3-induced *Cc*Caspase-A2 and *Cc*Caspase-B activation. However, *Cc*MINK1 did not affect *Cc*NLRP3-mediated *Cc*Caspase-A1 activity. Furthermore, we further examined the cleavage of *Cc*IL-1β. We found that the expression of mature *Cc*IL-1β mediated by *Cc*Caspase-A2 and *Cc*Caspase-B was induced in *Cc*MINK1 overexpressed group. However, *Cc*MINK1 had no effect on *Cc*Caspase-A1-mediated cleavage of *Cc*IL-1β (**Figures 5D–F**). These findings collectively indicate that *Cc*MINK1 plays a regulatory role in *Cc*NLRP3 inflammasome activation, specifically enhancing *Cc*Caspase-A2/B-dependent signaling pathways.

### 3.7 *Cc*MINK1 promotes *Cc*GSDME-mediated pyroptosis

**Figure 6:**
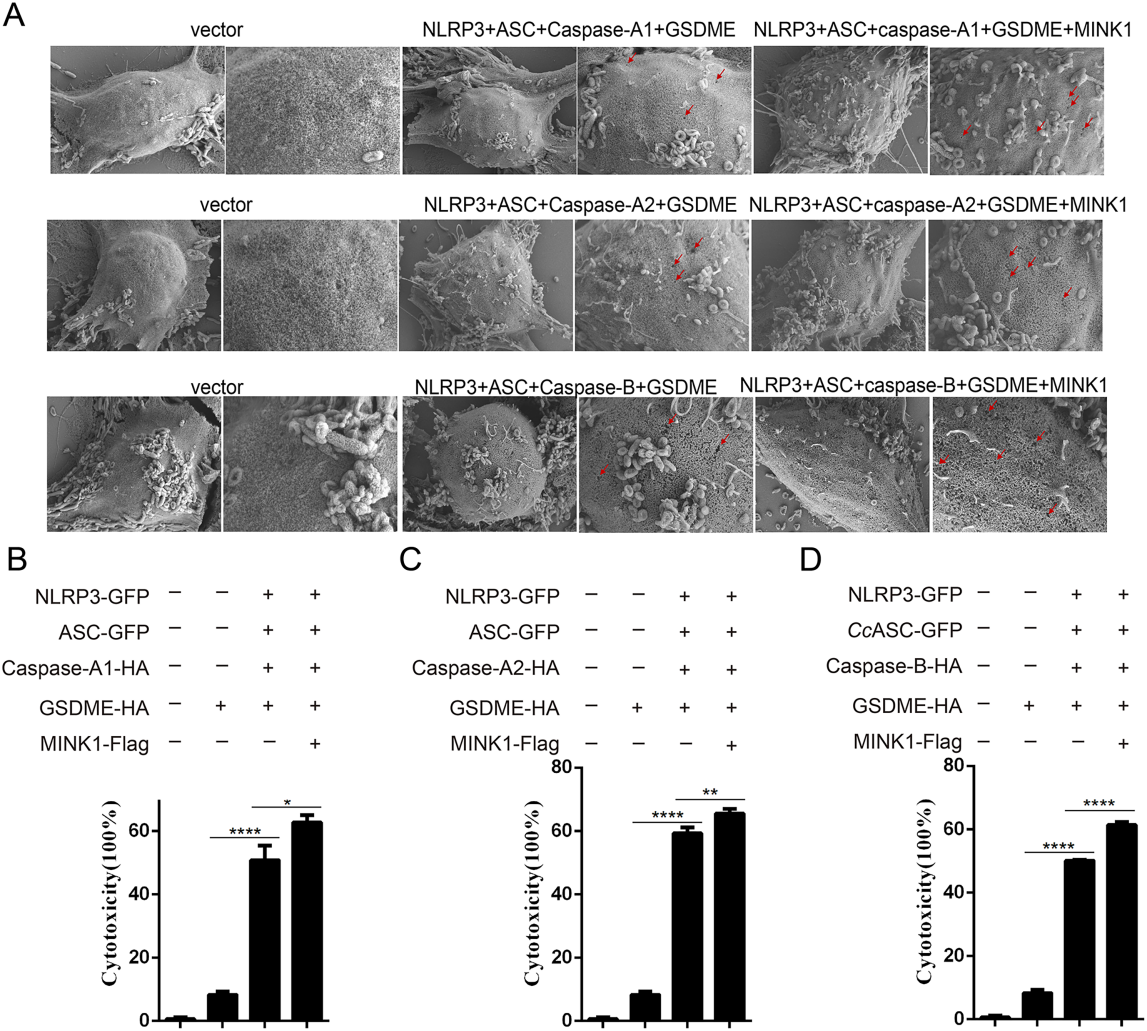
*Cc*MINK1 promotes *Cc*GSDME-mediated pyroptosis. **(A)** 293T cells were transfected with indicated plasmids. After 48 h, microscopic images of 293T cells were observed. Red arrows indicate the membrane pores induces *Cc*GSDME. **(B-D)** Indicated expression vectors were co-transfected into 293T cells. After 48 h, the supernatant was collected to detect LDH release. Data were emerged as mean ± SD (n=3) with at least three replicates, *P < 0.05, **P < 0.01 and ****P < 0.0001.

To investigate the role of *Cc*MINK1 in *Cc*GSDME-mediated pyroptosis, the recombinant plasmids of *Cc*MINK1, *Cc*GSDME and *Cc*NLRP3 inflammasome complex were co-transfected into 293T cells, which showed membrane pores and cell membrane rupture phenomenon on the cell membrane, and this phenomenon was more pronounced after overexpression of *Cc*MINK1 (**Figure 6A**). Additionally, the PI staining was applied to assess the level of pyroptosis. The results showed that the number of positively stained cells significantly increased in *Cc*MINK1 overexpressed cells compared to the control group (**Supplementary Figures 3A–C**). At the same time, the LDH release was increased significantly upon overexpression of *Cc*MINK1 compared with only inflammasome proteins (**Figures 6B–D**). Taken together, these results demonstrate that *Cc*MINK1 play a critical role in the *Cc*NLRP3 inflammasome-induced pyroptosis.

### 3.8 *Cc*MINK1 promotes *Cc*NLRP3 oligomerization and phosphorylation

**Figure 7:**
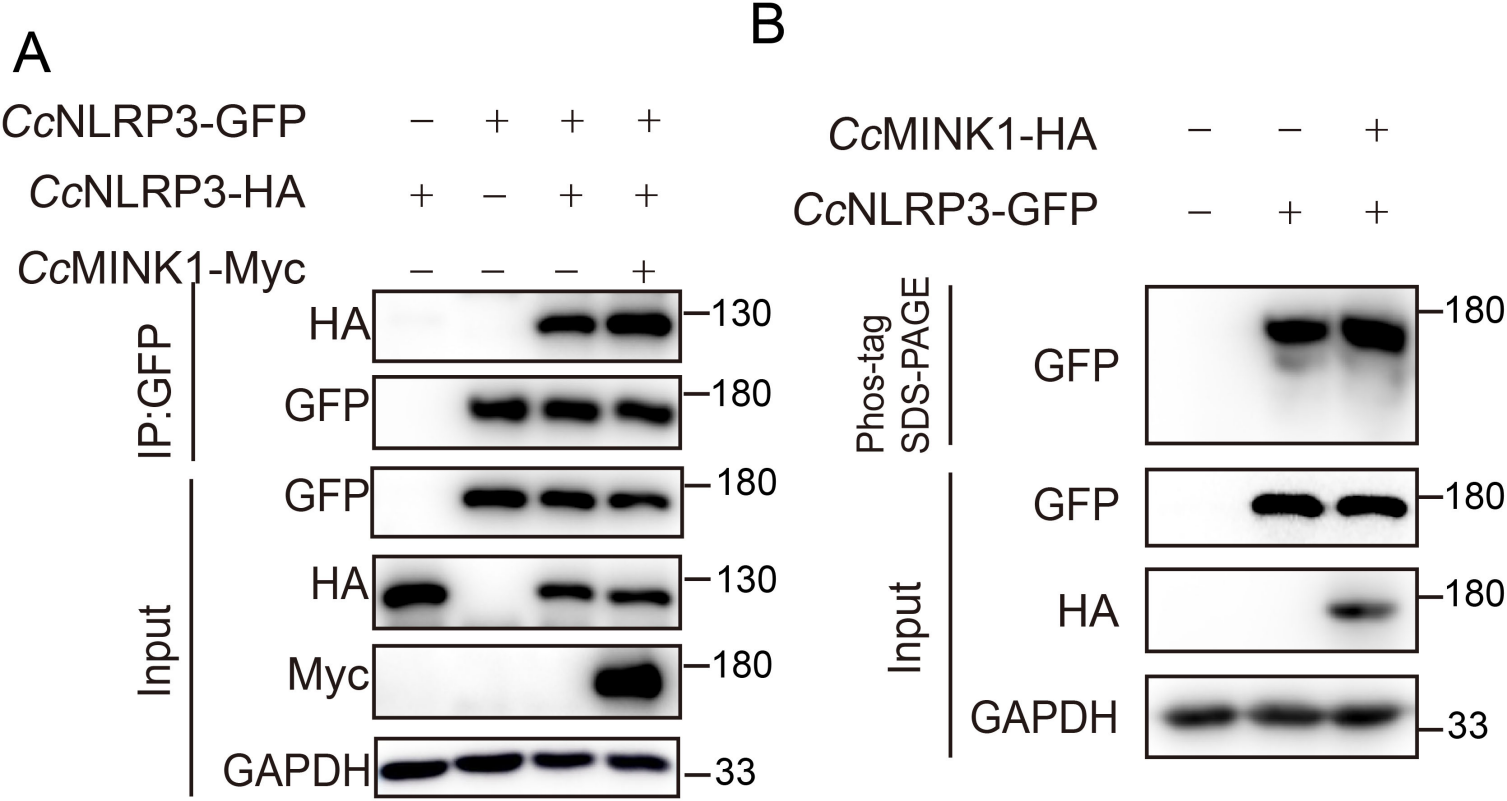
*Cc*MINK1 mediates the oligomerization and phosphorylation of *Cc*NLRP3. **(A)** 293T cells were transfected with *Cc*MINK1-Myc, *Cc*NLRP3-GFP and *Cc*NLRP3-HA expression plasmids. After 48 h, immunoprecipitation was performed with anti-GFP beads, and the input and IP samples were analyzed by immunoblot assays. **(B)** 293T cells were transfected with *Cc*MINK1-HA and *Cc*NLRP3-GFP. After 48 h, cell lysis were analyzed and Phos-tag SDS-PAGE and immunoblot assays were performed.

NLRP3 oligomerization and phosphorylation is critical steps in inflammasome assembly and activation (37). Hence, to determine the molecular mechanism by which *Cc*MINK1 promotes *Cc*NLRP3 activation, we examined whether *Cc*MINK1 affected *Cc*NLRP3 oligomerization and phosphorylation. *Cc*MINK1-Myc, *Cc*NLRP3-GFP and *Cc*NLRP3-HA was cotransfected into 293T cells, and Co-ip results showed that the *Cc*MINK1 promoted *Cc*NLRP3 self-interaction (**Figure 7A**). Additionally, phos-tag SDS-PAGE was used to determine whether *Cc*MINK1 phosphorylated *Cc*NLRP3. The result showed that the overexpression of *Cc*MINK1 significantly enhanced *Cc*NLRP3 phosphorylation compared to transfection of *Cc*NLRP3 alone (**Figure 7B**). Collectively, these results suggest that *Cc*MINK1 could induce the oligomerization and phosphorylation of *Cc*NLRP3.

### 3.9 The S_TKC domain of *Cc*MINK1 is required for *Cc*NLRP3-mediated inflammasome activation

**Figure 8:**
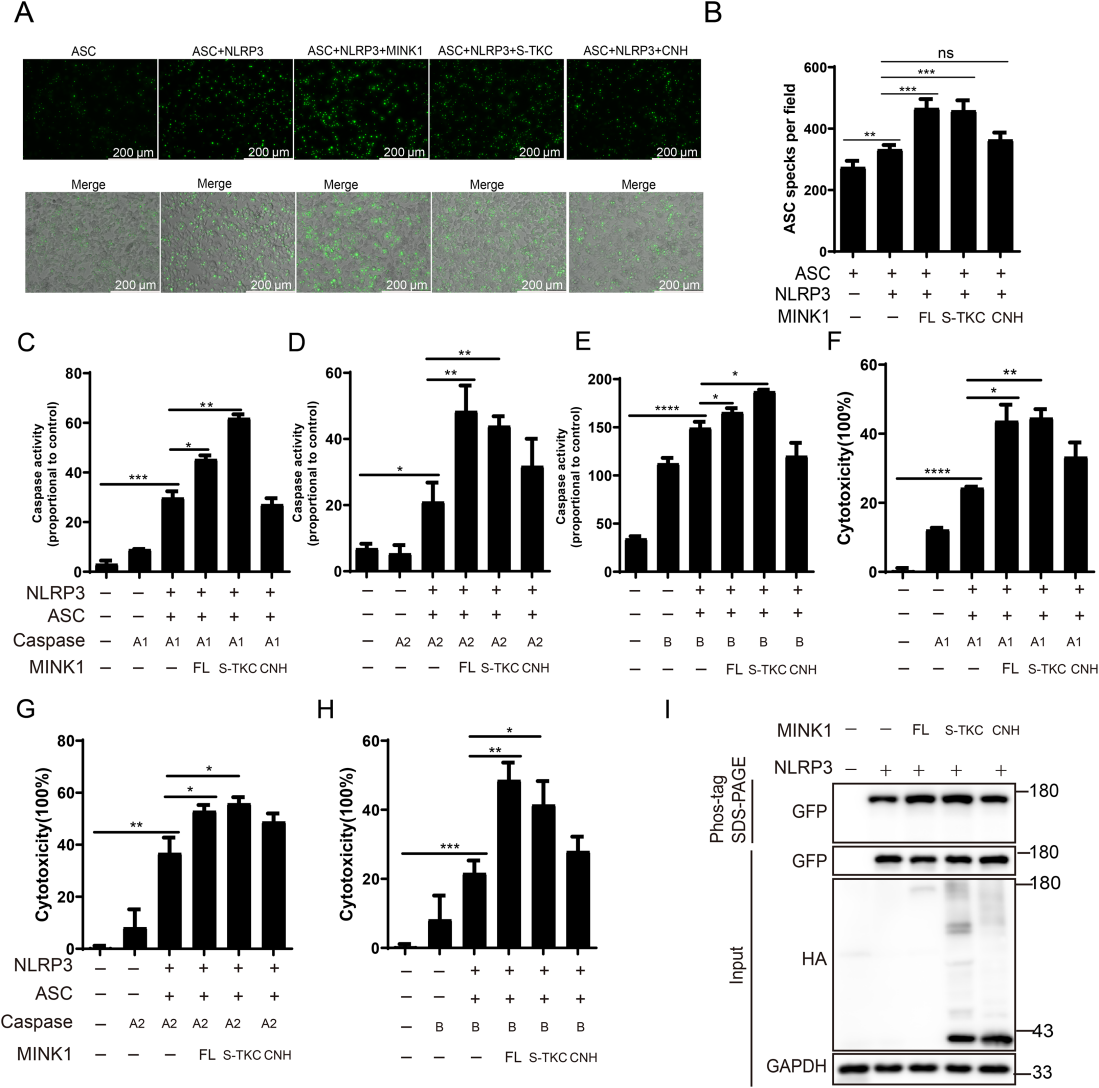
The S_TKC domain of *Cc*MINK1 is required for *Cc*NLRP3 inflammasome activation. **(A)** 293T cells transfected with the indicated plasmids. After 24 h transfection, the cells were observed and the images were visualized by confocal microscopy. **(B)** The relative mean value of *Cc*ASC specks in per field were calculated by ImageJ. **(C-E)** 293T cells were transfected with indicated plasmids. After 48 h, the activity of *Cc*Caspase-A1 **(C)**, *Cc*Caspase-A2 **(D)** and *Cc*Caspase-B **(E)** were examined. **(F-H)** *Cc*NLRP3-GFP, *Cc*ASC-GFP, *Cc*MINK1-HA and *Cc*Caspase-A1-HA **(F)**, *Cc*Caspase-A2-HA **(G)** and *Cc*Caspase-B-HA **(H)** vectors were co-transfected into 293T cells. After 48 h, the supernatant was collected to detect LDH release. **(I)** 293T cells were co-transfected with HA-tag full-length, S_TKC domain or CNH domain of *Cc*MINK1-HA and *Cc*NLRP3-GFP. After 48 h, cell lysis were analyzed and Phos-tag SDS-PAGE and immunoblot assays were performed. Data were emerged as mean ± SD (n=3) with at least three replicates, *P < 0.05, **P < 0.01, ***P < 0.001 and ****P < 0.0001.

Previous studies have shown that MINK1 exhibit several functions that are highly dependent on the kinase activity (38). Thus, we investigated whether *Cc*MINK1 regulates *Cc*NLRP3-mediated inflammasome activation via its kinase activity. We transfected full-length and domain mutants of *Cc*MINK1 into 293T cells. As shown in **Figures 8A, B**, the full length and S_TKC domain of *Cc*MINK1 significantly enhanced the speck formation of *Cc*ASC. However, the CNH domain of *Cc*MINK1 showed no such effect. Additionally, overexpression of full length and S_TKC domain of *Cc*MINK1, but not CNH domain, significantly enhanced *Cc*NLRP3-mediated *Cc*Caspase-A1, *Cc*Caspase-A2 and *Cc*Caspase-B activity (**Figures 8C-E**) and LDH release (**Figured 8F-H**). Meanwhile, phos-tag SDS-PAGE analysis revealed that the full-length *Cc*MINK1 and S_TKC domain mutants both increased *Cc*NLRP3 phosphorylation (**Figure 8I**). Taken together, these results suggest that S_TKC domain of *Cc*MINK1 is necessary and sufficient for promoting *Cc*NLRP3 inflammasome activation.

## 4 Discussion

As the most extensively characterized NLR family member, NLRP3 plays a pivotal role in orchestrating immune responses and maintaining inflammatory homeostasis. However, aberrant activation of the NLRP3 inflammasome is associated with the development of autoimmune and chronic inflammatory (39, 40). Hence, the activation of NLRP3 has to be tightly supervised and regulated to prevent host damage. While multiple regulatory mechanisms have been well-characterized in mammals (41), there is still limited in teleost. In the present study, we characterized *Cc*MINK1 as a novel regulator of *Cc*NLRP3 inflammasome activation. We found that *Cc*NLRP3 was phosphorylated by *Cc*MINK1, which was essential for *Cc*ASC oligomerization and inflammasome activation. And this process was dependent of its kinase activity. This study could provide new insights into the evolutionary conservation of inflammasome regulation across vertebrates.

The tissue expression analysis indicates that *Cc*MINK1 is highly expressed in the brain, hindgut, foregut and spleen of normal common carp. This may be closely related to the roles of these tissues in immune surveillance and barrier function. After stimulation by SVCV or *A. hydrophila*, the expression of *Cc*MINK1 in multiple tissues shows time-dependent changes. The expression of *Cc*MINK1 in the spleen, hindgut and foregut increases at 24 hours or 48 hours under viral stimulation, while the expression in the liver, hindgut and foregut significantly increases at 6 hours after bacterial stimulation. Under viral stimulation, the changes in gene expression are more complex and diverse, and there is a significant increase in multiple tissues in the later stage; however, bacterial stimulation causes the gene expression in multiple tissues to increase in the early stage. These results reflect that *Cc*MINK1 gene could play an important role in the innate immune response of common carp in dealing with bacterial and viral infections, but there are differences in the way and timing of activation of innate immunity by bacteria and viruses.

Post-translational modifications, which include phosphorylation, polyubiquitination, palmitoylation, and SUMOylation, play crucial roles in regulating NLRP3 activation through the direct targeting of NLRP3 as well as the downstream adaptors (42, 43). Phosphorylation serves as a critical molecular switch governing inflammasome activity, with distinct phosphorylation patterns either promoting or suppressing its assembly (42, 44, 45). For example, NIMA-related kinase 7 (NEK7), a mitotic kinase, facilitates NLRP3 inflammasome assembly and activation (46). JNK1-mediated NLRP3 phosphorylation is critical for NLRP3 deubiquitination, self-association and the subsequent inflammasome assembly (37). However, the protein kinase A (PKA)-induced phosphorylation of NLRP3 conversely inhibits nigericin-induced inflammasome activation (47). In the present study, we found that *Cc*MINK1 interacted with and phosphorylated *Cc*NLRP3. Additionally, phosphorylation modifications at different sites of NLRP3 may play distinct roles (47, 48). For example, Bruton’s tyrosine kinase (BTK) phosphorylates NLRP3 at polybasic linker between the PYD and NACHT domain and alters the net charge, that promotes NLRP3 translocation from the intact Golgi to the dTGN and inflammasome activation (49). In addition, phosphorylation mediated by BTK occurs within the FISNA domain, which facilitates conformation changes resulting from K^+^ efflux upon NLRP3 activation (50). Thus, the further clarification of the role and key domain and sites of *Cc*MINK1 in the *Cc*NLRP3 inflammasome is necessary.

MINK1 is a critical regulator of diverse physiological and pathological processes, including cellular growth, cytoskeletal rearrangement, motility, hemostasis and pyroptosis (51–53). Researchers have rarely reported whether and how MINK1 regulates inflammatory signaling and inflammasome activation. Recently, Zhu et al. reported that MINK1 could phosphorylate NLRP3 and positively regulate NLRP3 inflammasome signaling (29). Similarly, in our present study, *Cc*NLRP3 was found to be phosphorylated by *Cc*MINK1, which promoted *Cc*NLRP3 oligomerization and enhanced inflammasome assembly. In mammals, only the LRR domain of NLRP3 is capable of binding with MINK1. However, our results demonstrated that *Cc*MINK1 specifically interacted with the FISNA, NACHT, and PRY-SPRY domains of *Cc*NLRP3. The protein domains of mammals and fish NLRP3 are compared and found that there is a FISNA domain present mostly in fish NLRPs and a B30.2 (PRY-SPRY) domain unique in fish NLRs (54, 55), which may play key roles in the anti-infection immunity of fish. Our findings collectively reveal both conserved and species-specific mechanisms of *Cc*MINK1-mediated *Cc*NLRP3 regulation. However, the underlying mechanisms by which each domain functions still require further investigation.

In summary, we demonstrated that *Cc*MINK1 was a key regulator of the *Cc*NLRP3 signaling pathway. *Cc*MINK1 interacts with *Cc*NLRP3 and promotes its phosphorylation, leading to *Cc*NLRP3-mediated inflammasome assembly and activation. These results provide basic data for further study of inflammasome assembly in teleost, and theoretical basis for the prevention and treatment of fish infectious diseases.

## Data Availability

The original contributions presented in the study are included in the article/**Supplementary material**. Further inquiries can be directed to the corresponding author.

## Supplementary material

Supplementary Figure 1 The bioinformatic analysis of common carp MINK1 (*Cc*MINK1). **(A)** The schematic diagram of predicted *Cc*MINK1 protein structure was shown. **(B)** The spatial structure prediction of *Homo sapiens* MINK1, *Mus musculus* MINK1, *Danio aesculapii* MINK1, and *Cc*MINK1 protein using SWISS-MODEL. **(C)** Phylogenetic tree of MINK1 from carp and other species. The amino acid sequence of MINK1 was aligned via the neighbor-joining algorithm in MEGA 7.0 to generate a phylogenetic tree. The confidence of each branch was calculated through 1000 bootstrap replicates.

Supplementary Figure 2
*Cc*MINK1 augments *Cc*ASC specks formation in EPC cells. **(A)** EPC cells were transfected with the indicated plasmids. 24 hours post transfection, cells were observed, and the images were visualized by confocal microscopy. **(B)** The relative mean value of *Cc*ASC specks in per field were calculated by ImageJ. Data were emerged as mean ± SD (n=3) with at least three replicates, * *P* < 0.05, ** *P* < 0.01 and **** *P* < 0.0001.

Supplementary Figure 3
*Cc*MINK1 promotes cell pyroptosis. *Cc*Caspase-A **(A)**, *Cc*Caspase-A2 **(B)**, *Cc*Caspase-B **(C)** and *Cc*GSDME, *Cc*NLRP3, *Cc*ASC, *Cc*MINK1 were co-transfected into 293T cells. At 48 h, the cells were fixed with 4% PFA and PI staining was used. Then, the cells were visualized via rotary confocal microscope.
