# Supplementary figures and images for: Study on the regulatory role of MINK1 gene in the activation of NLRP3 inflammasome in common carp (Cyprinus carpio L.)
Source: Front Immunol. 2025 Oct 22;16:1663527. doi: 10.3389/fimmu.2025.1663527 (PMC12586150; doi:10.3389/fimmu.2025.1663527)

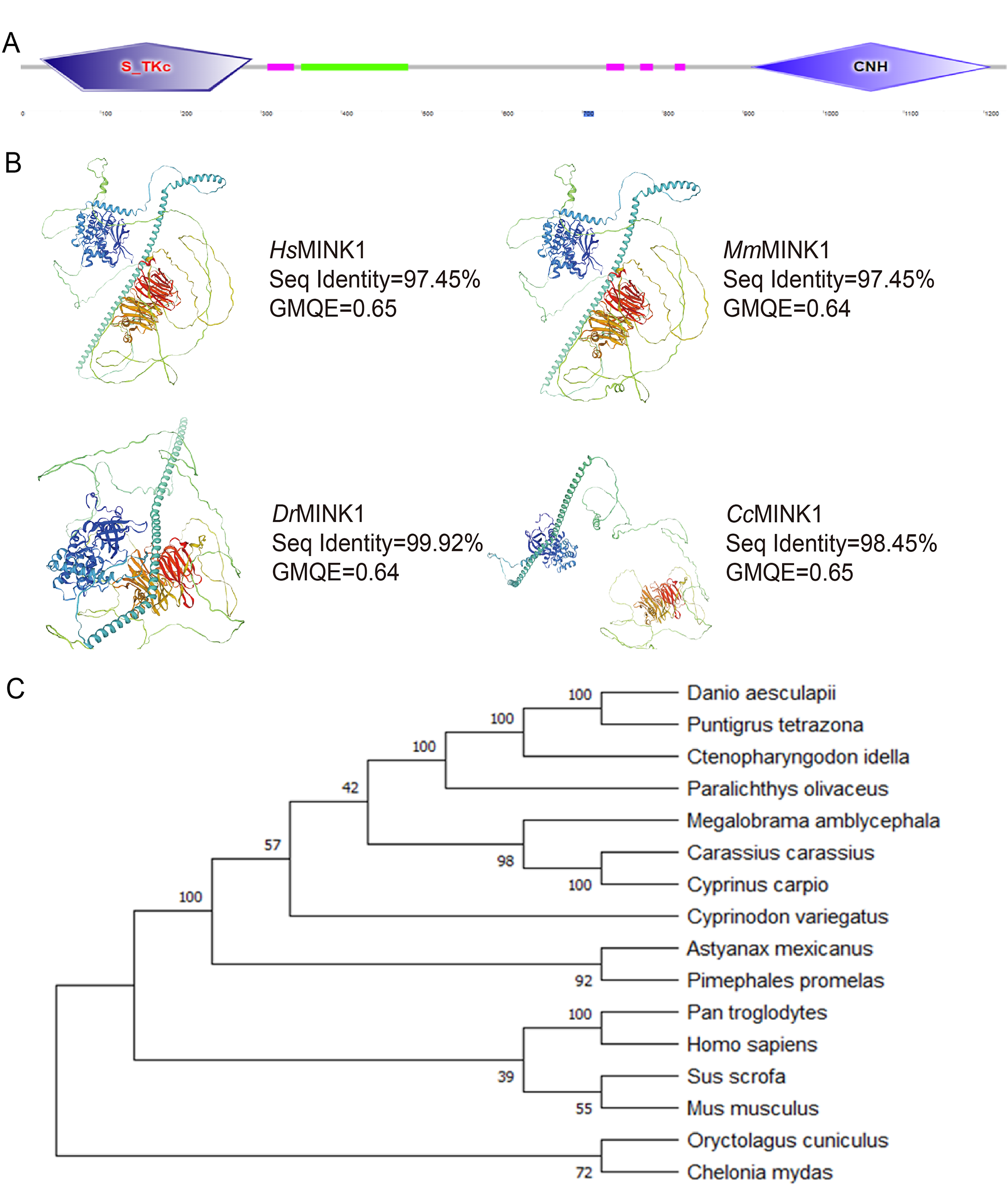

Supplement: Supplementary Figure 1 — The bioinformatic analysis of common carp MINK1 (CcMINK1). (A) The schematic diagram of predicted CcMINK1 protein structure was shown. (B) The spatial structure prediction of Homo sapiens MINK1, Mus musculus MINK1, Danio aesculapii MINK1, and CcMINK1 protein using SWISS-MODEL. (C) Phylogenetic tree of MINK1 from carp and other species. The amino acid sequence of MINK1 was aligned via the neighbor-joining algorithm in MEGA 7.0 to generate a phylogenetic tree. The confidence of each branch was calculated through 1000 bootstrap replicates. [file Image1.tif]

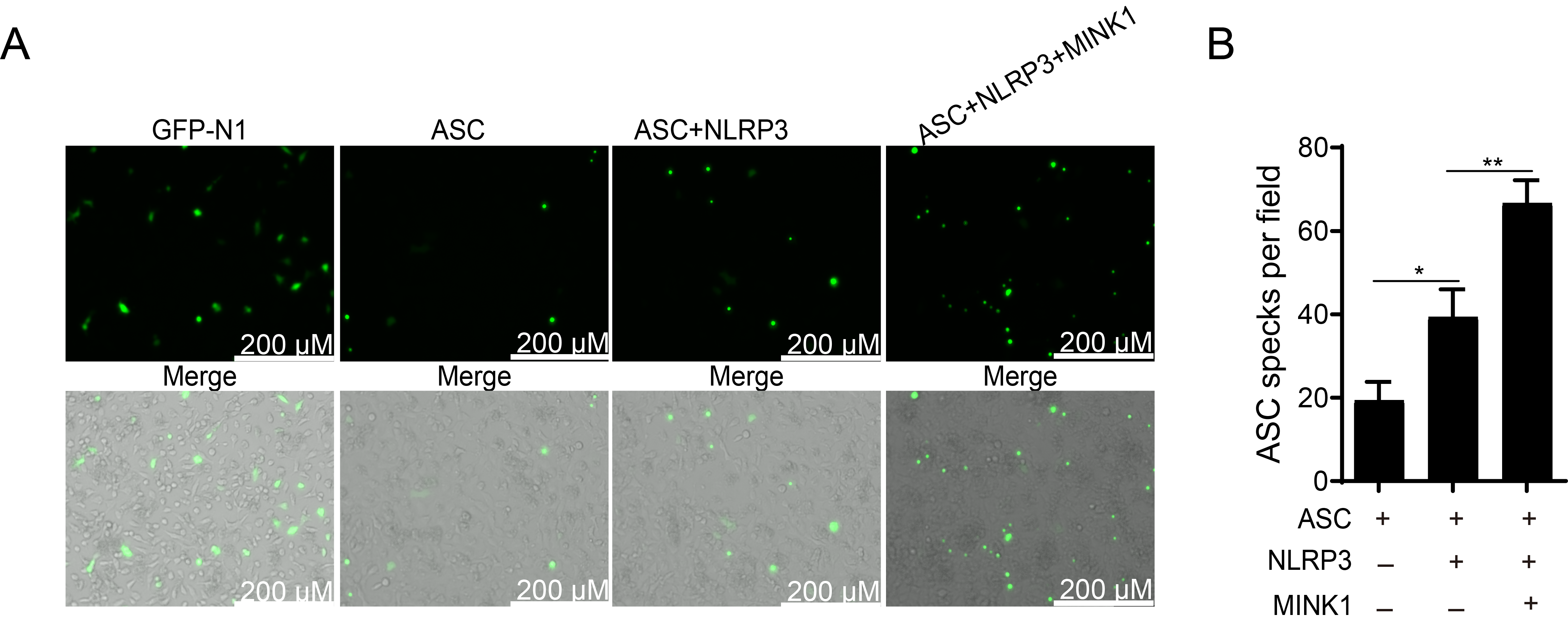

Supplement: Supplementary Figure 2 — CcMINK1 augments CcASC specks formation in EPC cells. (A) EPC cells were transfected with the indicated plasmids. 24 hours post transfection, cells were observed, and the images were visualized by confocal microscopy. (B) The relative mean value of CcASC specks in per field were calculated by ImageJ. Data were emerged as mean ± SD (n=3) with at least three replicates, * P < 0.05, ** P < 0.01 and **** P < 0.0001. [file Image2.tif]

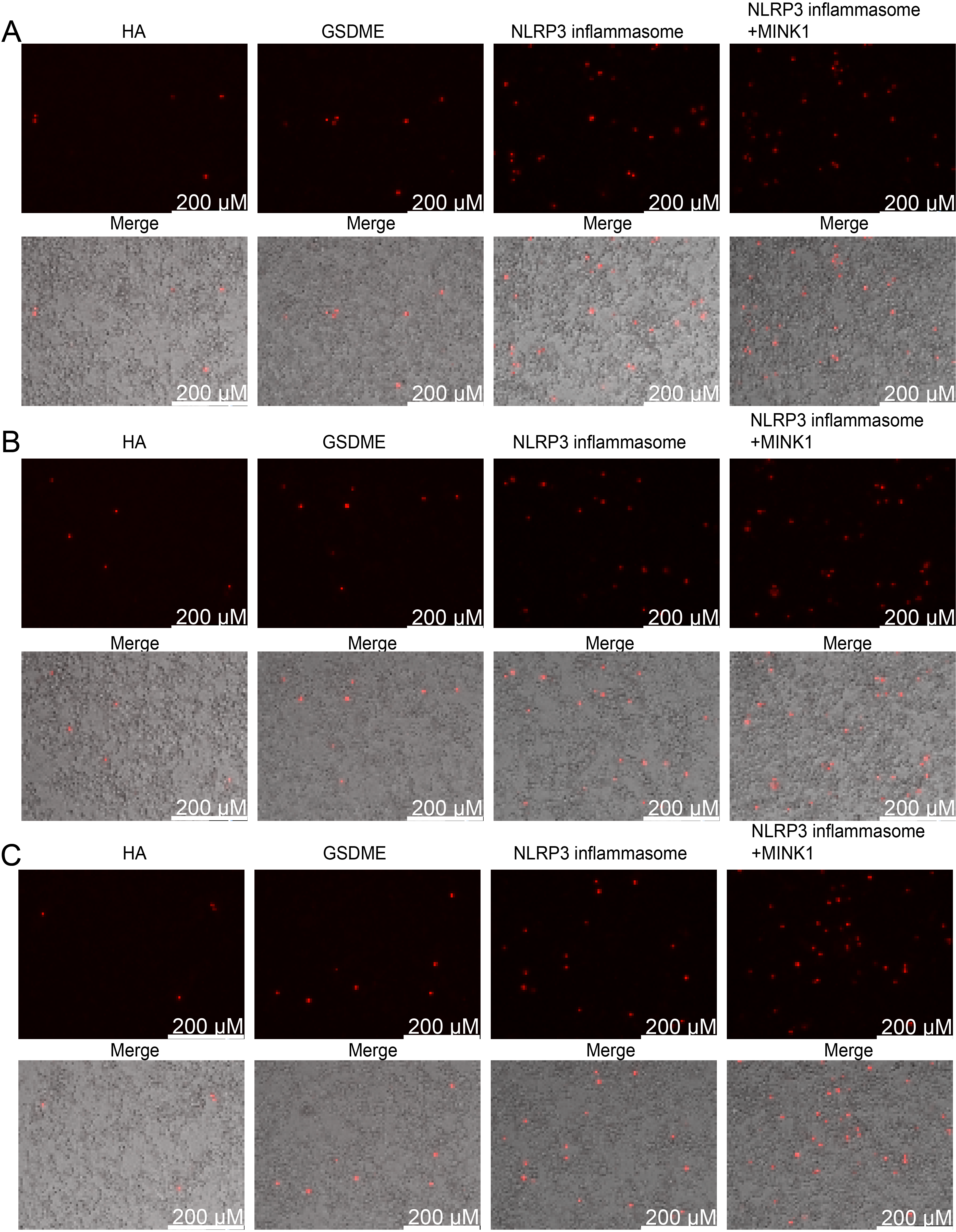

Supplement: Supplementary Figure 3 — CcMINK1 promotes cell pyroptosis. CcCaspase-A (A), CcCaspase-A2 (B), CcCaspase-B (C) and CcGSDME, CcNLRP3, CcASC, CcMINK1 were co-transfected into 293T cells. At 48 h, the cells were fixed with 4% PFA and PI staining was used. Then, the cells were visualized via rotary confocal microscope. [file Image3.tif]
